# The wellness incentives and navigation project: design and methods

**DOI:** 10.1186/s12913-015-1245-x

**Published:** 2015-12-29

**Authors:** Elizabeth Shenkman, Keith Muller, Bruce Vogel, Sara Jo Nixon, Alexander C. Wagenaar, Kimberly Case, Yi Guo, Martin Wegman, Jessie Aric, Dena Stoner

**Affiliations:** Department of Health Outcomes and Policy, College of Medicine, the Clinical and Translational Science Institute, and the Institute for Child Health Policy, University of Florida, Gainesville, USA; Department of Psychiatry, College of Medicine, University of Florida, Gainesville, USA; Department of Health Outcomes and Policy, College of Medicine and the Institute for Child Health Policy, University of Florida, Gainesville, USA; Department of Health Outcomes and Policy, College of Medicine, University of Florida, University of Florida, Gainesville, USA; Department of Health Outcomes and Policy, College of Medicine, Department of Epidemiology, College of Public Health and Health Professions, and the Clinical and Translational Science Institute, University of Florida,, Gainesville, USA; Texas Department of State Health Services, Austin, USA

**Keywords:** Patient Activation, Chronic Illness, Motivational Interviewing, Patient directed wellness

## Abstract

**Background:**

About 35 % of non-elderly U.S. adult Medicaid enrollees have a behavioral health condition, such as anxiety, mood disorders, substance use disorders, and/or serious mental illness. Individuals with serious mental illness, in particular, have mortality rates that are 2 to 3 times higher as the general population, which are due to multiple factors including inactivity, poor nutrition, and tobacco use. 61 % of Medicaid beneficiaries with behavioral health conditions also have multiple other co-occurring chronic physical health conditions, which further contributes to morbidity and mortality. The Wellness Incentives and Navigation (WIN) project is one of 10 projects under the Centers for Medicare and Medicaid Services “Medicaid Incentives for the Prevention of Chronic Diseases” Initiative, to “test the effectiveness of providing incentives directly to Medicaid beneficiaries of all ages who participate in prevention programs, and change their health risks and outcomes by adopting healthy behaviors.”

**Methods/Design:**

WIN is a three-year randomized pragmatic clinical trial designed to examine the comparative effectiveness of the combined use of personal navigators, motivational interviewing, and a flexible wellness account on cardiovascular risk reduction among individuals in Medicaid with co-occurring physical and mental health conditions or serious mental illness alone relative to the usual care provided within Medicaid Managed Care. 1250 individuals, identified through Medicaid claims data, were recruited and randomly assigned to an intervention group or control group with outcomes tracked annually. A comparison group was also recruited to help assess the study’s internal validity.

**Discussion:**

The primary outcomes are physical and mental health related quality-of-life as measured by the SF-12, and BMI, blood pressure, LDL-C, and Hba1c results for those who are diabetic measured clinically. The purpose of this paper is to present the unique design of the WIN trial prior to results becoming available in hopes of assisting other researchers in conducting community-based randomized pragmatic trials. Outcomes will be assessed through the linkage of patient reported outcomes, health care claims, and electronic health record data.

**Trial Registration:**

NCT02440906

## Background

Medicaid is the largest source of health care financing for individuals with behavioral health conditions. About 35 % of all non-elderly U.S. adult Medicaid enrollees have a behavioral health condition, such as anxiety, mood disorders, substance use disorders (SUD), and/or serious mental illness (SMI). SMI includes conditions such as schizophrenia, bipolar disorder, and major depressive episodes. Among those Medicaid beneficiaries with behavioral health conditions, 61 % also have a co-occurring chronic physical health condition, the majority of which include cardiovascular disease (CVD) and conditions linked to increased CVD morbidity and mortality, such as diabetes and chronic obstructive pulmonary disorder [1]. Individuals with SMI, in particular, are two to three times as likely to die from CVD as adults relative to the general population [2]. This high CVD prevalence and excess mortality, which contributes to increased health care use and expenditures, is often attributable to an increased occurrence of modifiable risk behaviors including inactivity, poor nutrition, and tobacco use [1, 3]. While some behavioral interventions yield moderately positive findings in terms of weight reduction and improved health related quality of life (HRQOL), significant gaps in knowledge remain about effective CVD risk reduction strategies among individuals with behavioral health conditions in general and those enrolled in Medicaid specifically [3].

For example, few CVD risk reduction studies include individuals with co-occurring physical and behavioral health conditions, especially those who have serious mental illness (SMI), despite their high risk for cardiovascular disease [3]. Moreover, most studies do not conduct multi-year follow-up of participant outcomes, limiting the available knowledge about long-term effects of behavioral interventions on CVD risk reduction [4, 5]. Finally, many CVD risk reduction studies are not embedded in real-world settings where individuals routinely receive their health care, limiting generalizability of the study findings.

We sought to address limitations of prior CVD risk reduction studies through the implementation and evaluation of the Wellness Incentive and Navigation (WIN) project. The WIN Project is one of 10 national demonstration projects funded across different states under the Centers for Medicare and Medicaid Services “Medicaid Incentives for the Prevention of Chronic Diseases (MIPCD)” Initiative. The goal of the initiative is to “test the effectiveness of providing incentives directly to Medicaid beneficiaries of all ages who participate in MIPCD prevention programs, and change their health risks and outcomes by adopting healthy behaviors” [6].

The WIN project is a three-year longitudinal randomized pragmatic clinical trial designed to examine the comparative effectiveness of the combined use of personal navigators, motivational interviewing (MI), and a flexible wellness expense account on CVD risk reduction among individuals in Medicaid with (1) co-occurring physical and mental health conditions or (2) SMI alone, relative to usual care provided within Medicaid Managed Care. The WIN intervention consists of (1) personal navigators housed within Medicaid managed care plans who work with participants to develop and refine health promotion goals and strategies using MI techniques; and (2) a person-directed flexible wellness expense account that provides participants with the financial infrastructure to purchase supplies and services to implement their wellness strategies (e.g. gym memberships; bicycles). The WIN Project is unique among the 10 state projects in its focus on individuals with co-occurring physical and behavioral health conditions, including SMI, its implementation through Medicaid Managed Care plans, the development of individualized person-centered goals, and the use of person-directed flexible wellness accounts. The person-centered goals were constrained to wellness behavior including improved nutrition and exercise to promote weight loss, tobacco cessation, and stress reduction, all known risk factors for CVD [3].

The purpose of this paper is to describe the WIN project in detail including the study’s (1) conceptual framework, (2) design, (3) participant recruitment, and (4) pragmatic focus.

### Conceptual framework

**Fig. 1:**
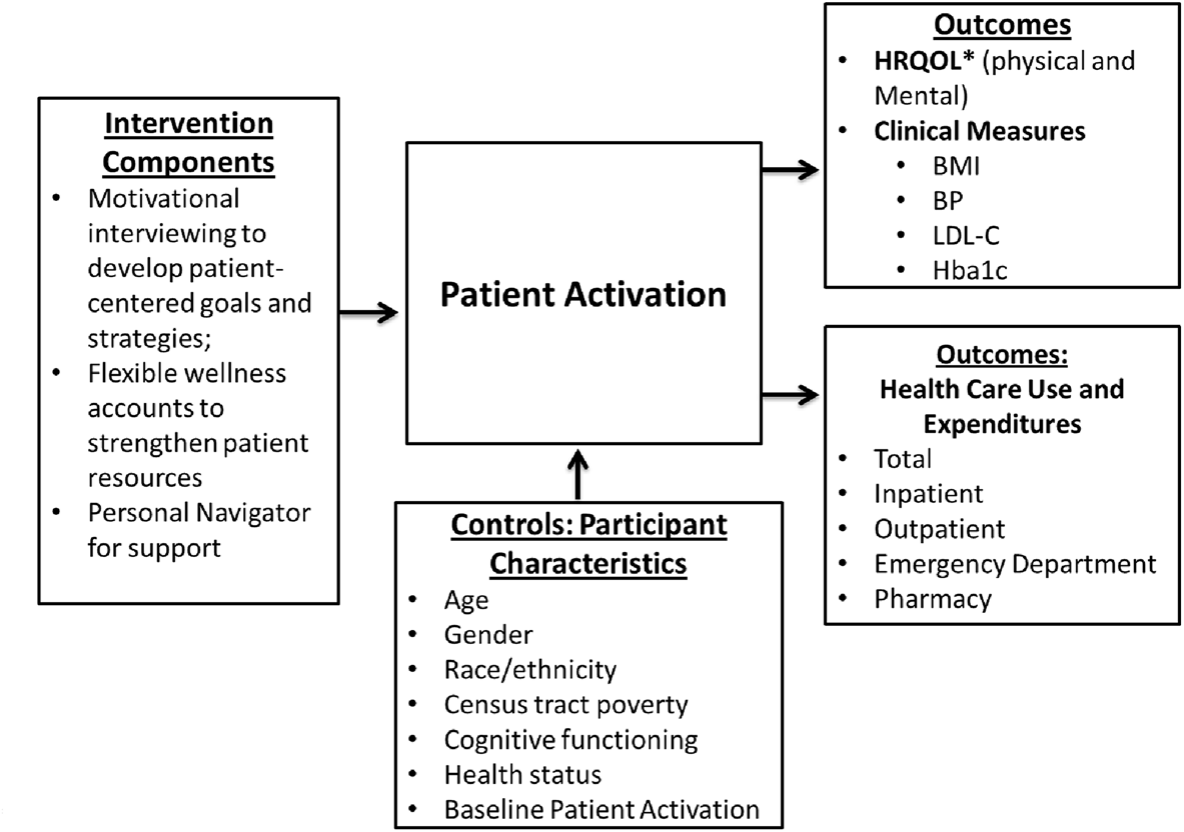
WIN Conceptual Frameworks

The WIN project combines three specific strategies, each of which has demonstrated effectiveness in facilitating health promotion activities to reduce CVD risk: personal navigators, motivational interviewing (MI), and consumer-directed wellness accounts, among healthy individuals and those with chronic conditions. However, to our knowledge, the effectiveness of these interventions has not been tested, in combination, with individuals with co-occurring physical and behavioral health conditions, including SMI. Our focus in the use of these strategies is rooted in the concept of patient activation (Fig. 1). Patient activation is defined as: “understanding one’s own role in the care process and having the knowledge, skills, and confidence to take on that role”. Patients who are more activated, as measured by the Patient Activation Measure (PAM) [7], engage in more positive health behaviors including: disease self-management [8], better diet and exercise [9], preventive care, and seeking and using health information [10].

In our conceptual framework, we hypothesize that patient activation mediates the effects of the combined intervention components to: (1) increase participant health related quality of life (HRQOL), (2) improve the clinical measures of body mass index (BMI), blood pressure (BP), Low Density Lipoprotein- Cholesterol (LDL-C), and Hemoglobin a1c (Hba1c), and (3) reduce health care use and expenditures (total, inpatient, outpatient, emergency department, and pharmacy). In our conceptual framework, we also incorporate six categories of control variables that may influence an individual’s capacity to take an active role in managing their health and experience increased patient activation (participant age, gender, race/ethnicity, census tract poverty, cognitive functioning, and health status) [11]. We hypothesize that these control variables will have an influence on patient activation and also exert a direct effect on the outcomes of interest.

Health coaches called personal navigators have been shown to increase patient activation and contribute to a range of health outcomes including improved blood pressure and lipoprotein levels and reduced emergency department visits and hospitalizations [7]. MI is a person-centered counseling approach used to help individuals explore and resolve their ambivalence around behavior change in favor of positive behavioral changes [12]. The MI approach initially demonstrated efficacy in treating individuals with substance use disorders (SUDs). MI-based health coaching also has been shown to increase activation levels and perceived overall health status among individuals with chronic conditions, such as diabetes, heart failure, chronic obstructive pulmonary disease (COPD), and mental illness. Individuals with chronic conditions receiving MI-based health coaching demonstrate increased readiness to make lifestyle changes related to drug, alcohol and tobacco use, physical activity, stress management, weight management and sleep [13, 14].

Medicaid recipients often do not have the financial resources to take action on their own initiative to make nutrition, exercise, and lifestyle changes to improve their own health. There is a small, but growing, body of evidence about the use of financial resources to help individuals make lifestyle changes to improve their health. Typical financial intervention studies involve rewarding participants with a financial incentive for achieving health goals, such as smoking cessation and weight loss. Funds are not usually provided to support the purchase of items or services to help the participant achieve his or her goals. Financial incentives have some demonstrated effectiveness in helping participants achieve their goals compared with control groups not receiving incentives, although the outcomes are mixed and tend to be short term [15–21].

In contrast to traditional financial incentive models, consumer-directed approaches provide participants with the autonomy and the funds to direct portions of their own care and to purchase health services that are meaningful to them. Consumer-directed efforts are considered particularly important for individuals with chronic conditions to better support them in managing their chronic conditions within the context of their functional status and living conditions [22, 23]. Contextual characteristics may support or constrain an individual’s ability to increase their activation and play a more active role in their health and health care. Access to financial resources influences an individual’s ability to acquire the skills to improve their health and may have an effect on the levels of patient activation [10]. While the effects of providing financial resources to increase patient activation have not been specifically tested, to our knowledge, increasing financial resources allows participants to purchase items that will enhance their ability to manage their health.

For the WIN Project, the use of personal navigators, MI, and flexible funds were integrated to form the intervention. The purpose of the intervention is to foster the achievement of health goals in a Medicaid population with co-occurring physical and mental health conditions or SMI through increasing patient activation. Cross-sectional analyses of adult patients seen in a large health system have shown that for every additional 10 points in patient activation, using the Patient Activation Measure (PAM), the predicted probability of having an ED visit, being obese, or smoking decreased by one percentage point. Further, the likelihood of having clinical indicators in the normal range (Hba1c and LDL-C) was one percentage point higher [9]. It is not known whether these same changes will be observed in a population with co-occurring physical and mental health conditions.

For the WIN intervention, personal navigators use MI to collaborate with the participants to address the discrepancy between their current and desired health behaviors. The WIN personal navigators then support autonomous choice by helping the participants identify strategies or options that can be used to resolve those discrepancies [24]. The participants are given a flexible wellness account of up to $1150 annually for up to three years to purchase services and supports to implement the strategies they identified to meet their health goals.

Contextual variables also influence patient activation including socio-demographic and health characteristics [11]. The relationship between cognitive functioning and patient activation is not known. However, issues related to cognitive functioning may have a significant effect on capacity to engage with personal navigators, establish and meet goals, and manage a flexible wellness account [25]. Deficits in executive functioning, in particular, which includes planning, organizing, strategizing, paying attention to and remembering details, and managing time, have been linked to poor health outcomes and inadequate self-care [26, 27].

Age, gender, race/ethnicity, and health status are associated with worse health outcomes. For example, older adults and racial/ethnic minorities are more likely to report poorer health status and to have hypertension, obesity, high cholesterol, and poor Hba1c control than younger, non-minority individuals [28–31]. They also are more likely to have potentially preventable emergency department visits and hospitalizations [30, 32–34]. Further, individuals residing in census tracts with high percentages of families residing below 100 % of the federal poverty level (FPL) are more likely to experience adverse health outcomes [34, 35].

In keeping with a person-centered approach, our primary study outcomes are participant self-reported physical and mental HRQOL; and changes in clinical measures that are associated with cardiovascular health including: BMI, BP, and total cholesterol (LDL-C) results as well as Hba1c results for those who are diabetic. We hypothesize, consistent with the findings of other studies, that participants will have improved HRQOL and clinical measures.

Secondarily, we are examining whether patient activation is a mediator related to changes in inpatient, emergency department (ED), outpatient, pharmacy and total health care expenditures for the WIN participants. We hypothesize that total health care expenditures will be reduced because the participants have higher patient activation and greater capacity to manage their health.

Expenditures also will be examined relative to the cost of running the WIN Program, providing estimates of the cost-effectiveness of the program. While our analysis is not specific to expenditures associated with CVD, a broad expenditure analysis will provide important information that can be used to determine if the program is financially sustainable.

## Methods

### Study design

This study is a longitudinal randomized pragmatic clinical trial (PCTs) with randomization at the individual Medicaid enrollee level. Individuals were randomized to an intervention group to receive the combined intervention components of (1) personal navigators professionally trained in motivational interviewing and (2) flexible wellness accounts or to a control group receiving the usual care provided within Medicaid managed care. The study recruitment procedures are described more fully under Recruitment Strategies.

All members meeting the WIN criteria eligibility criteria were randomly assigned to the intervention or control group prior to seeking consent to participate. This technique, known as a Zelen design, is utilized in research trials in which there is a large difference in services provided to the treatment group relative to the control group [36]. This design technique eliminates the disappointment of the control group in not receiving the benefits provided to the treatment group, thereby reducing resentful demoralization and attrition and improving internal validity of the design. Following the Zelen design concept, after the individual is randomized, the potential participant is contacted and only receives an explanation of the intervention that he or she will receive for the arm in which he or she was assigned. The study recruitment procedures are described more fully under Recruitment Strategies.

### Study setting

The three Medicaid Managed Care Plans participating in the Texas STAR + PLUS Program in Houston, Texas were invited to participate – Amerigroup, UnitedHealth Care, and Molina Health Plan. The STAR + PLUS Program began in 1997 in Houston as a 1915(b)/(c) waiver program for individuals in Medicaid with disabilities severe enough to qualify for supplemental security income. Program waivers authorized by sections 1915(b) and 1915(c) of the Social Security Act are “intended to control costs, while allowing states administrative flexibility to operate their programs” [37]. Section 1915(b) waivers permit states to waive the freedom of choice provision so that eligible beneficiaries receive health care services from a limited set of providers; typically within a managed care network model. Section 1915(c) authorizes states to provide home- and community- based services (HCBS) to individuals who might otherwise require care in an institutional setting such as a nursing home. HCBS can include personal attendant services, homemaker services, case management, environmental modifications, and respite care.

Overall, the primary focus of the STAR + PLUS Program is to improve the quality of care for enrollees with disabilities through coordinated and comprehensive care. The program delivers acute and long-term services through a single system, employs service coordinators who develop individual care plans and assist enrollees in receiving health care services, and emphasizes HCBS as alternatives to institutional care [38]. As of 2014, STAR + PLUS is offered throughout Texas. The Harris service delivery area (SDA) was selected for the WIN Project because Harris County was the site where the STAR + PLUS Program began. Two of the three Harris SDA plans have been involved since the inception of STAR + PLUS. The managed care plans’ longevity and experience with the program in the Harris SDA provided sufficient infrastructure and stability to launch a pragmatic clinical trial, with personal navigators hired within each of the managed care plans.

A comparison group, not receiving the intervention, was also recruited to assess the external validity of the study. The comparison group of STAR + PLUS enrollees reside in two adjacent communities: Corpus Christi, San Antonio and the immediate surrounding counties, which comprise the Nueces and Bexar SDAs and otherwise have the same criteria for WIN eligibility as the intervention and control groups. The individuals were randomly selected from among the managed care plans participating in STAR + PLUS in those areas; which were the same managed care plans participating in the WIN Study in Houston. The comparison group will provide enhanced information about whether any changes in the outcomes are attributable to the effects of the intervention.

### WIN as a pragmatic clinical trial

In designing WIN as a pragmatic clinical trial, the research team used the Pragmatic-Explanatory Indicator Summary (PRECIS) instrument, which is an instrument designed to assist researchers in clarifying and refining the study along a pragmatic-exploratory continuum [39]. PCTs are designed to estimate the effectiveness of interventions under usual, real-world conditions in health care applications [40]. In contrast, efficacy or explanatory studies examine interventions under ideal clinical conditions. The PRECIS instrument was developed because “very few trials are purely pragmatic or explanatory” but rather vary along a continuum within key domains [41]. Table 1 describes how the WIN project fits within the 10 domains of the PRECIS instrument.

**Table 1:** The WIN Project Design using the PRECIS Domains

Domain	WIN Design
Eligibility criteria for participants	Trial enrollment limited to individuals with co-occurring physical and behavioral health conditions and/or SMI, and current enrollment in the Texas Medicaid STAR + PLUS Program in the Harris Service Delivery Area. The strict enrollment criteria used are typically seen in explanatory trials.
Extent of flexibility in application of the intervention	The combination of personal navigators, use of MI, and the $1150 annual flexible wellness account is prescribed and must be followed. However, the self-directed nature of the intervention components reflects a participant-centered, tailored intervention. The intervention is pragmatic in that the navigators were embedded in Medicaid managed care plans were health care is normally delivered and the health and wellness goals and strategies were developed based on participant preferences.
Degree of personal navigator expertise in applying and monitoring the intervention	All personal navigators underwent an initial 1.5-day MI training by a psychologist who is an expert in MI. Following this training, navigators participated in 5 weeks of constructive phone calls addressing specific taped interactions. The personal navigators also undergo annual MI refresher training. An expert psychologist also monitors 2 randomly selected appointments using the Motivational Interviewing Treatment Integrity (MITI) coding scheme to assess navigator competency and adherence to MI on 6 dimensions (Global Rating, Giving Information, Questions, Simple Reflections, Complex Reflections, Spirit). The psychologist then provides feedback and refresher information to the personal navigators if their scores fall below minimal requirements for competency. The navigators adapt their approach to their participants’ needs but are required to follow MI techniques. This component of WIN has both pragmatic and explanatory elements.
Extent of flexibility in application of standard care for the control group	The standard care provided to the control group represents the usual care provided through the Medicaid Managed Care Plans. The State has contractual requirements for the Medicaid Managed Care Plans but the Plans have latitude in how they meet those requirements. This component of WIN is pragmatic.
Degree of practitioner expertise in applying and monitoring the application of standard care for the control group	Each Medicaid Managed Care Plan has a provider network and the providers must meet a set of licensure and state standards to be part of the network. However, the practitioners likely vary widely in how care is delivered to the Medicaid enrollees. This component of WIN is pragmatic.
Intensity of follow-up of trial participants	Trial participants are monitored closely through continued contact with study personnel. Intervention participants are contacted monthly by navigators and if not successfully contacted, a number of retention efforts are planned to re-engage this member back into the trial. Control and comparison participants are contacted monthly by mail and phone to provide updated contact information. This component of WIN is explanatory.
Nature of the primary outcomes	The primary outcomes are participant reported physical and mental health related quality of life using the SF12, which is measured in a standardized format; and clinical variables including blood pressure; HDL, LDL-C, Hba1c results for those who are diabetic; and BMI, which are obtained from the participants’ medical records. In addition, inpatient, emergency department, outpatient and total health care expenditures are obtained from the Medicaid claims and encounter data. The primary outcome is collected using methods consistent with an explanatory trial. The medical record and health care expenditure results are pragmatic in that the information is gathered for purposes other than the WIN Project.
Participant protocol compliance	The participants meet in-person with their personal navigators once every three months and have monthly teleconference calls where wellness goals and strategies are reviewed and discussed. Participants who have not completed appointments are contacted in a number of ways to ensure continued and timely completion. The participants are monitored monthly in terms of the spending on their flexible wellness accounts. Participants who are not using their flexible wellness accounts as planned receive additional support from the personal navigator in purchasing items to support their wellness goals. This component of WIN is explanatory.
Navigator protocol compliance	In addition to the monthly monitoring of MI-adherence, navigators are closely monitored for the number of participants successfully completing appointments each month. Navigators falling below recommended study standards are further consulted to discuss barriers for success. This component of WIN is explanatory.
Specification and scope of analysis of primary outcomes	The analysis plan was pre-specified and models follow an intent-to-treat structure to alleviate the bias in randomization of participants.

The WIN project varies along the pragmatic-explanatory continuum in each of the domains. Ultimately, the WIN research team characterized the study as a PCT because of the following key characteristics: (1) the personal navigators are embedded within Medicaid managed care plans, which are the usual source of care for individuals meeting the study eligibility criteria; (2) the personal navigators are monitored in conducting the intervention but the person-centered nature of the intervention leads to adaptability to individual needs; (3) participants are monitored for their adherence to the wellness strategies they selected through in-person meetings and teleconferences with the navigators, but they have flexibility in choosing their wellness goals and strategies; and (4) the Medicaid managed care plans provide care to the control group according to contractual obligations with the State of Texas Medicaid Program but also according to their own internal standards, which can vary among the plans.

### Participants

#### Inclusion criteria

STAR + PLUS enrollees in Amerigroup, United, or Molina Health Plans, age 21–55, in the Harris SDA in Texas were eligible to participate. Diagnostic inclusion criteria consisted of: (1) serious mental illness (SMI) diagnosis (e.g. schizophrenia, bi-polar disorder, major depressive disorder) alone; (2) behavioral health diagnoses (e.g., anxiety, depression, substance use disorder) coupled with a chronic physical health diagnosis (e.g. diabetes, COPD); or (3) all three categories – SMI, behavioral health diagnosis and a chronic physical health condition. Table 2 shows the diagnostic criteria for classification of SMI, behavioral health conditions, and physical health chronic conditions.

**Table 2:** Diagnostic Criteria for Participation in the WIN Study

Variable Name	Diagnostic Criteria
Physical Health Condition (PHC)	One or more of the following ICD-9 codes present in any field (e.g., primary, secondary) Asthma 493. xx; Cerebrovascular Disease 430.xx-438.xx; Intracranial Injury (excluding skull fracture) 851.xx-854.xx; Chronic Obstructive Pulmonary Disease and Allied Conditions 490.xx-496.xx; Neoplasms 140–239; Diabetes 250.xx; Heart Failure 428.xx; HIV/AIDS 042, V08, 079.53, 795.71; Hereditary and Degenerative Diseases of the Central Nervous System 330.xx-337.xx; Other Disorders of the Central Nervous System 340.xx-349.xx; Disorders of the Peripheral Nervous System 350.xx-359.xx; Rheumatoid Arthritis and Other Inflammatory Polyarthropathies 714.xx; and Hepatitis (alcoholic 571.1; history of hepatitis C v12.09; hepatitis type C 070.51, 070.54, 070.70.
Serious Mental Illness (SMI)	One or more of the following ICD-9 codes present in any field (e.g., primary, secondary) – measuring serious mental illness: Schizophrenia 295.xx; Episodic Mood Disorders 296.xx; Delusional Disorders 297.xx; Other Non-Organic Psychoses 298.xx
Behavioral Health Conditions (BHC)	Neurotic Disorders, Personality Disorders, and Other Non-Psychotic Mental Disorders (Includes alcohol and drug dependence syndromes) (300.xx-316.xx).

#### Exclusion criteria

Individuals were excluded if they were dually eligible for Medicare and Medicaid or if they had intellectual or cognitive diagnoses indicative of severe cognitive impairment including: 290.X (dementia) and 318–319 (moderate to severe mental retardation). These individuals were excluded because of concerns that they could not provide informed consent or adequately participate the intervention. The participants were identified using Medicaid enrollment files and health care claims data from Calendar Year 2011, provided through a data use agreement with the Texas Health and Human Services Commission.

#### Recruitment procedures

Individuals meeting the inclusion and exclusion criteria were randomly selected from the STAR + PLUS Harris SDA enrollment files to contact about potential study participation. The targeted number of intervention participants varied by the size of the managed care plan. Amerigroup and United HealthCare had similar STAR + PLUS enrollment sizes and Molina had the smallest. Therefore, the final targeted number of WIN Study participants per health plan was 500 each for Amerigroup and United HealthCare and 250 for Molina.

Consistent with the Zelen Design, individuals were assigned to either the intervention or control group prior to making contact with the individuals. Both groups were told that the WIN study was focused on understanding the best ways to help individuals with physical and mental health conditions and/or SMI develop and meet health and wellness goals that may improve their health outcomes including blood pressure and cholesterol control. Those randomly assigned to the intervention group received an explanation of the intervention strategies and information about baseline and annual telephone surveys. Those in the control and comparison group were told that they would participate in baseline and annual telephone surveys.

The primary recruitment method involved mailing an introductory letter customized to the individual’s group assignment and the recruitment brochure followed by telephone outreach within 7 to 10 days of mailing the letter. Participants were recruited in waves from May 2012 through July 2013 with the National Opinion Research Center (NORC) conducting the outreach calls. Recruitment progress was monitored through weekly calls with NORC with the goal of enrolling 40 total participants per week across the two larger plans and 20 participants per week in the smaller health plan.

For those individuals who were not able to be contacted via letter or phone, research assistants were provided a list of participant names and addresses and went door-to-door providing the introductory letter and brochure with the goal of obtaining updated contact information and/or consent to participate in the study. N = 150 door-to-door outreach attempts were made, yielding 11 participants. Due to the low yield, the door-to-door outreach was discontinued after 1 month and ongoing effort was placed on the telephone contact.

**Fig.2:**
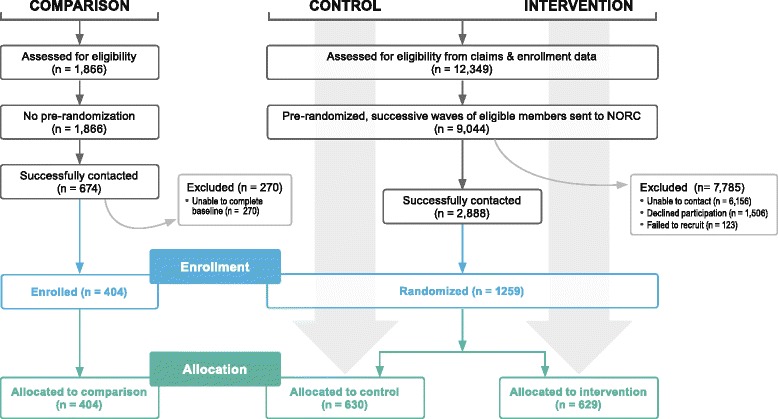
Sample selection procedures

For the intervention and control groups, 12,349 individuals were initially identified as meeting the WIN eligibility criteria in the Harris SDA and 9,044 randomly selected names from that group were sent to NORC for attempted contact; 2888 were successfully contacted; and 1259 agreed to participate in the study (Fig. 2). Analyses comparing those who agreed to be in either the control or the intervention group relative to those who could not be located or refused revealed no significant differences in race/ethnicity, or diagnostic category, though more females than males chose to participate (p < .001).

### Intervention

#### Personal navigators

A total of ten personal navigators were hired across the three plans – four each for Amerigroup and United HealthCare and two for Molina. Each personal navigator has a caseload of approximately 70 participants. The intervention was designed to allow for two in-person meetings, held one month apart, in the participant’s home. Following these initial meetings, the participant receives two monthly telephone calls and a quarterly in-person meeting. The caseload size was selected to balance the estimated amount of time each navigator would spend on each intervention group participant during a 40 hour work week along with cost considerations related to the number of navigators that could be funded through the project. Approximately 1 hour is allocated for each intervention participant per month to review and discuss goals, progress toward those goals, and the identification of wellness strategies and associated purchases using the flexible wellness account. An additional hour was provided to account for travel to the participants’ homes, contact with the participant and other documentation paperwork.

#### Motivational interviewing

The personal navigators use MI techniques when working with the intervention participants. The MI training and ongoing monitoring of the navigators’ MI techniques is described in Table 1. Using MI techniques, the Navigators work with the participants to identify their health risks and to understand their motivations for change. Emphasis is placed on identifying health risks, which participants were either acknowledging as an issue or already taking steps to change.

#### Flexible wellness accounts

Once the participant has identified strategies to meet his/her wellness goals, the participant and navigator discuss and select items to purchase. For example, a participant has identified obesity as a health risk, 20-pound weight loss as a goal, and stress reduction and increased physical activity in the form of walking to meet those goals. The navigator then works with the participant to identify services and/or items needed to meet these goals, such as books or recordings on mindfulness and stress reduction, walking shoes, and yoga classes. The necessary funds to purchase the items and services are placed on the participant’s debit card. Funds are added to the debit card as needed to purchase agreed upon items and services up to a maximum of $1150 annually for each of three years.

The debit card system is novel because participants are given financial support to meet their health goals. In traditional wellness programs, participants receive a financial incentive for completing a health goal or action (e.g. given $20 for losing 20 pounds). Traditional approaches may be constrained in their success among low-income populations that do not possess the financial means to purchase items for wellness. The navigators provide training to the participant in debit card use. In addition, participants sign agreements acknowledging that debit card funds may only be used for agreed upon purchases and that they are restricted from purchasing certain items (e.g., items/services covered by their health plan, vitamins, e-cigarettes). Participants are asked to provide receipts to their navigators at each in-person visit. The participants’ agree to allow the transactions to be monitored through the Bank of America, which provides the debit cards.

#### Visit schedule – intervention group

The intervention group visits are organized into three types: Intake, Wellness, and Annual Review. One in-home intake visit was held where the participant: 1) completes a health risk assessment tool, 2) discusses health risks identified from the tool, 3) develops goals to address selected risks, and 4) identifies strategies to meet those goals. The wellness visits consist of monthly phone calls and a quarterly in-home visit. During the wellness visits, the navigator reviews the participants’: health risks, goals, and strategies; self-reported outcomes in meeting the goals; wellness purchases made to address the goals; and self-reported satisfaction with strategies to reduce health risks and purchases made; and requests for new strategies and purchases to meet health goals. During the annual review visits, participants complete a new health risk assessment, review current progress and goals, and determine whether they wish to add or remove selected health risks, goals and strategies.

#### Control and comparison group interactions

The control and comparison groups complete the health risk assessment at baseline and annually. The control group is contacted on a monthly basis and the comparison group on a quarterly basis via mailings requesting current contact information. Monetary compensation is provided for completing the baseline and annual surveys and for maintaining monthly or quarterly contact. Due to the high transitive nature of this population, this strategy ensures retention of a high-risk population.

### Measures

The first primary outcome measure is self-reported physical and mental HRQOL using the Short Form-12 (SF-12). The SF-12 has been validated across a number of chronic diseases and conditions [42]. The survey consists of 12 questions measuring functional health and well-being. Participants answer questions related to daily functioning, difficulties in physical tasks, and disruptions in life due to mental illness (e.g. depression, anxiety). The overall score can be further classified into two summary scores for physical and mental health. We also are examining whether there are changes in the following clinical measures: 1) blood pressure, 2) BMI, 3) cholesterol (LDL-c), and 4) Hba1c (only for participants with diabetes). Secondary outcome measures are: health care expenditures reported as total, inpatient, outpatient and emergency department expenditures.

Participant reported HRQOL is assessed at baseline and annually for each of the three study years after entry. All other primary and secondary outcomes are assessed for two years prior to the participants’ entry into the WIN study and annually for each of three years thereafter. The clinical data are recorded from all outpatient encounters in the electronic and paper health record for the baseline year and for each of the subsequent three years. The expenditure data are obtained from the Medicaid health care claims data.

Health status, sociodemographic, and cognitive information are also collected. Health status is measured using the 3 M Clinical Risk Groups (CRGs), which uses ICD-9-CM diagnosis codes from health care claims to assign participants to hierarchically defined health status groups [43]. Participants are classified into the following health status categories: minor chronic conditions (e.g., asthma), moderate chronic conditions (e.g., diabetes), or major chronic conditions (e.g., cancer).

Sociodemographic control variables included age (in years), gender, and race/ethnicity (non-Hispanic white, non-Hispanic black, Hispanic, other). Contextual geographic variables included the percentage of the population living in poverty in the participants’ census tracts.

Another variable that may play an important but underappreciated role is cognitive ability, which is strongly related to clinical symptoms and overall functioning [44]. Patients’ educational level is often applied as a proxy for cognitive ability. However, education may not reflect current capacity though it may reflect prior achievement. This dissonance may be particularly evident in chronically ill populations such as those participating in the WIN study, for which medications, uncontrolled disease, and life stressors may adversely affect brain integrity and cognitive abilities [45–47]. Thus, accounting for cognitive capacity may be a critical control variable when examining intervention outcomes.

A cognitive battery previously developed by Wilson and colleagues (2005, 2010) was used to gather information about the participants. This battery was developed to examine key components of executive function, specifically attention, semantic organization and working memory. These processes are interrelated and are commonly presumed to underlie the effective conduct of daily life requirements. The individual tests are briefly described below and administered following approaches used in previous work [48, 49].

To assess the ability to encode novel logically-related information, participants listened to a tape of the “Anna” story taken from the Wechsler Memory Scale [50]. Immediately following the tape, participants are asked to recall as much of the story as possible. Standard administration and scoring guidelines are used.

To assess attention and working memory, participants complete three digit span tasks. In the forward and backward digit span tests, interviewers read sets of digits (of increasing lengths) at a set pace of 1/sec. Set length increases to 8 (forward) or 7 (backward) digits or until participants respond incorrectly to two sets of the same length. Participants are required to repeat the digits in the order presented (forward) or to invert the order (backward). In the digit ordering test, interviewers read a list of digits which may include repeated numbers. Participants are required to recall the digits in their appropriate numerical sequence and to recall duplicates of a number. For example, if the interviewer read “4, 2, 8, 2, correct recall would be reflected in the response “2, 2, 4, 8”. Set length increases to 9 digits or until participants respond incorrectly to two sets of the same length.

The ability to manipulate information in existing semantic networks was assessed with two fluency tests. The first test requires participants to recall as many animals as possible in one minute, the second, vegetables. Proper nouns and repeated items are scored as incorrect. Fantasy animals (e.g., unicorns) are also disregarded. Certain fruits commonly classified as vegetables, such as tomatoes were accepted.

### Data sources

This study requires linking health care claims data with person-reported outcomes, executive functioning results, and electronic or paper health record data. Person-level administrative enrollment and claims data provided by Texas Health and Human Services Commission is being used to obtain the participants’ age, sex, race/ethnicity, and census tract of residence. Enrollment records are linked to claims data that includes International Classification of Diseases (ICD-9-CM) diagnosis codes, Current Procedural Terminology (CPT) codes, charges, National Drug Codes (NDC), rendering provider, billing provider, and dollar amount of claim paid to the provider, The rendering provider information is used to identify the participants’ primary care providers to obtain electronic health records. Electronic and paper health records are used to abstract the clinical data (e.g., blood pressure, BMI, LDL-c, and Hba1c). These data will be supplemented with county-, Zip Code Tabulation Area- and census tract- level data from the U.S. Census Bureau, and U.S. Department of Commerce in order to capture important geographic contextual factors.

### Ethical considerations

The University of Florida Health Science Center Institutional Review Board (IRB-01) reviewed and approved all study materials including the study protocol, informed consent document, recruitment materials, and assessment instruments. Informed consent documents were obtained electronically from intervention participants and verbally from the control and comparison participants. The trial was registered with ClinicalTrials.gov.

### Sample size

We performed the power analysis based on the hypothesis test of whether there is a significant treatment by time interaction in the analysis of repeated measurements of the primary outcome of SF-12 scores. The SF-12 scores are measured a total of four times during the study period: baseline, year 1, year 2, and year 3. In order to align the power analysis with the primary data analysis [51], we computed statistical power based on a longitudinal model with SF-12 from study years 1 through 3 as responses and the baseline (year 0) measure as a covariate.

We estimated that the mean and standard deviation of the SF-12 to be 50 and 10, respectively [52]. Based on reports in the SF-12 manual and previous publications that used the SF-12, we estimated the reliability of the scale to be 0.70 across one year. For the test-retest correlation matrix, we assumed that it followed a generalization of an autocorrelation (AR) form called a LEAR model [53]. The LEAR model allows greater flexibility when modeling correlation decay across time. Finally, we used the Bonferroni correction to control the Type I error rate, which was set at 0.01 ÷ 2 since there were two SF-12 scales: physical and mental.

We used the power software POWERLIB [54] to produce a power curve, assuming the treatment and the control group each has a sample size of 625 participants. We expect the power to be 0.90 for detecting a 2.5-point difference in mean SF-12 score between the treatment and control groups.

### Statistical analysis

#### Descriptive analyses

We will calculate summary statistics for the demographic variables separately for intervention, control and comparison groups. Continuous demographic variables will be described as mean ± SD or median (25th percentile, 75th percentile). Categorical demographic variables will be described as percentages. We will follow an intent-to-treat (ITT) approach when performing data analysis.

#### Primary outcomes

The primary outcomes are the SF-12 scores and clinical measures including BMI, BP, LDL-C, and Hba1c results for those who are diabetic. All primary outcomes are measured at baseline, year 1, year 2, and year 3. For the clinical measures, yearly values will be calculated by taking a median of all available values. For each primary outcome, we will fit linear mixed models to analyze the repeated measurements using the PROC MIXED procedure in SAS, Version 9.4. In each model, the responses are the years 1 to 3 measures. Predictors include the outcome baseline measure, age, gender, race/ethnicity, census tract poverty, clinical risk group, treatment, as well as interactions of treatment-by-baseline, time-by-baseline, time-by-treatment, and time-by-treatment-by-baseline. We will also test estimable trends of time (linear and quadratic) in the model. We will fit an unstructured covariance across time and employ necessary model diagnostic procedures [55]. We will adopt a backwards selection strategy to remove insignificant predictors based on the Full Model in Every Cell, followed with added-in-order tests for all model terms involving treatment group or time [55].

#### Longitudinal mediation analysis

In addition to examining the treatment effect on the primary outcomes, we will also examine whether the treatment effect on the outcomes is mediated through patient activation (Fig. 1). Given the presence of longitudinal data, we will use an autoregressive mediation model to evaluate the mediation effect of patient activation. We will follow the guidelines in Mackinnon for model building and diagnostics [56]. The time-specific indirect effects and overall indirect effect of patient activation will be computed and evaluated for significance. This will address the question of whether patient activation mediates the effect of treatment on the primary outcomes at any time between waves 1 and 4, rather than at some specific point.

#### Secondary outcomes

The secondary outcomes are inpatient, outpatient, emergency department, pharmacy, and total expenditures. All secondary outcomes are also measured at baseline, year 1, year 2, and year 3. We are using the same linear mixed model analytic approach to analyze these outcomes. The planned secondary analyses differ from the primary analyses only in the choice of outcome variable. We will employ the same model selection and diagnostic procedures with appropriate attention to the peculiarities of cost distributions (probability masses at zero, heteroskedasticity, skewness, and kurtosis).

For the comparison group, 1,866 names were sent to NORC for attempted contact, 674 individuals were contacted and 404 agreed to participate in the study. The sociodemographic and diagnostic characteristics of the comparison group do not differ significantly from those of the intervention and control groups.

## Discussion

The WIN project is a randomized pragmatic clinical trial designed to promote health and wellness with the goal of reducing CVD risk among Medicaid enrollees with mental and/or physical health-related disabilities. This project has several unique features. First, the project focuses on individuals who are traditionally not included in clinical trials – those with co-occurring physical and mental health conditions and SMI. Second, the study focuses on increasing participant activation, which has been shown to result in improved health outcomes, through a unique combination of strategies: MI, personal navigators and a flexible wellness account. Third, the WIN project is unique in its use of a flexible wellness account as opposed to traditional financial incentives. Participants have the unique opportunity to make choices about what will work best for them to meet their health goals, with the guidance and support of a personal navigator. The participant-centered approach to goal setting, the development of strategies to meet those goals, and the opportunity to choose and pay for goods and services to implement the strategies is novel; particularly among this vulnerable population.

Fourth, the WIN project is embedded in a real world setting. The participating health plans employ the navigators and participate in collaborative monitoring with the study team on issues of navigator performance and internal fidelity. The information gained from conducting the study in a health plan setting will provide valuable information about the ultimate sustainability of the intervention beyond the study. If the health and expenditure outcomes are positive, state Medicaid Programs and their participating health plans will have detailed information about how to implement such a program based on the WIN experience.

Fifth, novel data integration strategies are used throughout the project. The STAR + PLUS Medicaid enrollment and claims/encounter data were used for cohort identification for the intervention, control, and comparison groups. Access to the statewide STAR + PLUS enrollment and claims data made the construction of a comparison group residing outside of the Harris Service Area (Houston) possible. Moreover, the linkage of participant reported outcomes about achievement of their health goals, electronic health record, and claims/encounter data provide a comprehensive picture of the study outcomes for this vulnerable population.

In summary, the novel design of this project and its participant-centered approaches applied to a rarely studied population will enhance our knowledge about strategies for CVD risk reduction among individuals with co-occurring physical and mental illness.

## Abbreviations

WIN: Wellness Incentive and Navigation
SMI: Serious Mental Illness
CVD: Cardiovascular disease
HRQOL: Health related quality of life
MIPCD: Medicaid Incentives for the Prevention of Chronic Diseases
MI: Motivational Interviewing
PAM: Patient activation measure
BMI: Body/Mass index
LDL: Low Density Lipoprotein- Cholesterol
Hba1c: Hemoglobin a1c
SUD: Substance use disorder
COPD: Chronic obstructive pulmonary disease
FPL: Federal poverty level
ED: Emergency department
PCT: Pragmatic clinical trial
HCBS: Home- and community- based services
SDA: Service delivery area
PRECIS: Pragmatic-Explanatory Indicator Summary
NORC: National Opinion Research Center
SF-12: Short Form 12
CRG: Clinical Risk Group
NDC: National Drug Codes
CPT: Current Procedural Terminology
ICD-9-CM: International Classification of Diseases
ITT: Intent-to-treat
